# Gender, power, and violence: A systematic review of measures and their association with male perpetration of IPV

**DOI:** 10.1371/journal.pone.0207091

**Published:** 2018-11-29

**Authors:** Katharine J. McCarthy, Ruchi Mehta, Nicole A. Haberland

**Affiliations:** 1 Population Council, New York, New York, United States of America; 2 Mailman School of Public Health, Columbia University, New York, New York, United States of America; University of Westminster, UNITED KINGDOM

## Abstract

**Introduction:**

Harmful gender norms, views on the acceptability of violence against women, and power inequities in relationships have been explored as key drivers of male perpetration of intimate partner violence (IPV). Yet such antecedents have been inconsistently measured in the empirical literature. This systematic review aimed to identify which measures of gender inequitable norms, views, relations and practices are currently being used in the field, and which are most closely tied with male IPV perpetration.

**Methods:**

We searched five electronic databases to identify studies published between 2000 and 2015 that reported the association between such gender inequities and male perpetration of IPV. Identified scales were categorized by content area and level of generality, as well as other attributes, and we compared the consistency of scale performance across each category.

**Results:**

Twenty-three studies were identified, employing 64 measures. Scales were categorized into three main thematic areas: views on gender roles/norms, acceptance of violence against women, and gender-related inequities in relationship power and control. We also classified whether the scale was oriented to respondents’ own views, or what they believed others do or think. While overall, measures were positively associated with IPV perpetration in 45% of cases, this finding varied by scale type. Measures inclusive of acceptance of violence against women or beliefs about men’s sexual entitlement, followed by scales that measured respondents’ views on gender roles/norms, were most consistently associated with IPV perpetration. Measures of relationship power showed less consistent associations. We found few scales that measured peer or community norms.

**Conclusion:**

Validated scales that encompass views on the acceptance of violence against women, and scales inclusive of beliefs about men’s sexual entitlement, may be particularly promising for unpacking pathways to IPV perpetration, targeting interventions, and monitoring progress in IPV prevention efforts. A number of gaps in the literature are identified.

## Introduction

Intimate partner violence (IPV) is a significant human rights and public health concern. Globally, an estimated 30% of ever-partnered women and girls have experienced physical or sexual IPV, with reported lifetime estimates as high as 71% among women in Ethiopia [1,2]. At the same time, a substantial proportion of men report perpetration of physical or sexual IPV. For example, population-based estimates from six countries in Asia and the Pacific documented lifetime estimates ranging between 25.4% of men in rural Indonesia to 80.0% in Bougainville, Papua New Guinea [3].

Activists, theorists, researchers and practitioners have articulated how intimate partner violence is both a product of, and helps perpetuate, a larger gender system (or gender order) [4–6]. This gender system generates and reinforces inequity which often gives men power over women through the distribution of resources, social norms, institutional practices, social interactions, patterns of behavior, and internalized beliefs and identities [7,8]. These factors operate across multiple levels, including societal, community, individual, and interactional, such as families, workplace and intimate relationships. Male perpetration of IPV is linked with multiple components throughout this gender system, including norms, views, practices and relations.

Socially constructed ideologies about masculinity–or the expectations and beliefs about what men should do or what attributes they should perform [9,10]–are implicated in men’s perpetration of violence. For example, masculinity ideology frequently includes roles and qualities such as strength, toughness, control, and sexual dominance that may be demonstrated through violence [4]. Social norms regarding IPV include descriptive norms–perceptions of what others do (e.g., beliefs about IPV frequency in the community or among peers)–and injunctive norms (e.g., beliefs about whether others approve or disapprove of IPV) [11,12]. At the interpersonal level, unequal power in relationships, for example, is enforced through violence or the threat of violence, as well as by controlling daily household decision-making and circumscribing a partner’s autonomy, aspirations, and access to social and economic resources [13]. Individual-level attitudes, beliefs and behaviors–for example, whether a person believes that physical violence against a wife is justified, or the degree to which a man endorses or adheres to masculine norms and roles–also contribute to whether he inflicts violence on a partner. While beyond the scope of this paper, gender inequities at the structural level–such as laws and policies that do not consider forced sex within marriage rape, or that place the burden of proof on the victim of partner violence–further weave IPV into the fabric and processes of a multi-level gender system.

The pathways between gender inequitable norms, views, practices, relations and IPV may be augmented and buttressed by other risk factors for IPV including exposure to violence in childhood, gang membership, substance use, low socioeconomic status, and unemployment [6,14–16]. These factors may operate directly on likelihood of IPV perpetration, or may influence other variables in the gender system [13]. For example, men may struggle to attain a masculine ideal of ‘provider’ when jobs are scarce, leaving few options for demonstrating masculinity other than through violence against other males and female partners [3,5]. Growing up in an abusive household which models aggression may also normalize violence, resulting in reinforcement of harmful masculinity norms and intergenerational replication of IPV [4,6,14].

The relationship between IPV and the components of the gender system has been well-documented. Recent large-scale, multi-country studies such as the International Men and Gender Equality Survey (IMAGES) and the UN Multi-Country Study on Men and Violence, for example, identify inequitable gender beliefs, permissive attitudes about violence against women, and controlling relationship practices as important risk factors for male perpetration of violence [3,17]. Specifically, the UN study, which involved more than 10,000 men in six countries of Asia and the Pacific, found that the two factors most associated with perpetration of both physical and sexual IPV were controlling behaviors and inequitable gender attitudes [3]. Attributable fraction values–or the proportion of IPV perpetration attributable to these factors–ranged from 6.7% to 10.5% across countries for controlling behaviors, and 20.4% to 23.4% for gender inequitable attitudes.

Given the potential of components of the gender system to condone and promote violence through multiple pathways, addressing them has emerged as a central component of IPV prevention efforts [4,14,18]. However, while some studies have demonstrated an association between gender inequitable norms, views, practices, or relations and IPV perpetration [19–23], others have not [24–27]. A limitation in understanding this association is that the definition and measurement of such constructs of gender inequity has varied, reflecting the large number of hypothesized pathways, but leading to an incomplete understanding of which variables and scales have the most explanatory power in predicting violence perpetration.

At a practical level, theories of change which underpin intervention design can also benefit from greater clarity in terms of what aspects of the gender system, i.e., gender inequitable norms, views, relations or practices, are most associated with IPV perpetration, and at what level of society (e.g., community, interpersonal (family, peer or intimate/sexual relationships), or individual) such constructs are most salient. A better understanding of how gender inequitable norms, views, relations and practices have been measured and what specific scales are most associated with IPV perpetration can inform effective intervention design by helping to target program content, platforms, and reach, as well as identify which scales may be best suited to monitor progress in IPV prevention efforts.

To address these questions, we conducted a systematic review of the published and grey literature to identify: (1) what measures of gender norms, views, relationships, and practices have been implemented in the field, and (2) which measures are most consistently associated with IPV perpetration. We empirically classify identified measures by scale content and referent or level of generality or (i.e., community, peer or individual-level) and synthesize what can be gleaned from existing evidence as well as what gaps remain in measurement and understanding.

## Methods

This systematic review follows the PRISMA guidelines [28].

### Article identification

Articles were identified using key term searches of five electronic databases: Pubmed, EconLit, SocIndex, POPLine and Women’s Studies International. Key terms included “gender norms”, “gender beliefs”, “gender inequity”, “gender relations”, “relationship power” and “women’s agency”. The full search string is listed in S1 Table. Hand searches of specific journals (e.g., *Gender and Development*, *Journal of Interpersonal Violence*, *Culture*, *Health and Sexuality*) were also performed to identify relevant titles. Reference lists of included studies were also searched resulting in the inclusion of three additional studies.

### Inclusion and exclusion criteria

Articles were included if they were peer reviewed, primary research published in English between January 2000 and August 2015, included participants ages 10 to 49, and reported a quantitative association between measures of gender inequity–i.e., gender inequitable norms, views, relations, and practices—and IPV perpetration. We considered studies which measured the association between males’ responses to these scales and male to female perpetration of IPV in heterosexual relationships. Studies were included if male reports of gender inequity were modeled as the independent variable and male IPV perpetration was the outcome. We excluded studies where it was not possible to locate or classify scale items.

**Fig 1:**
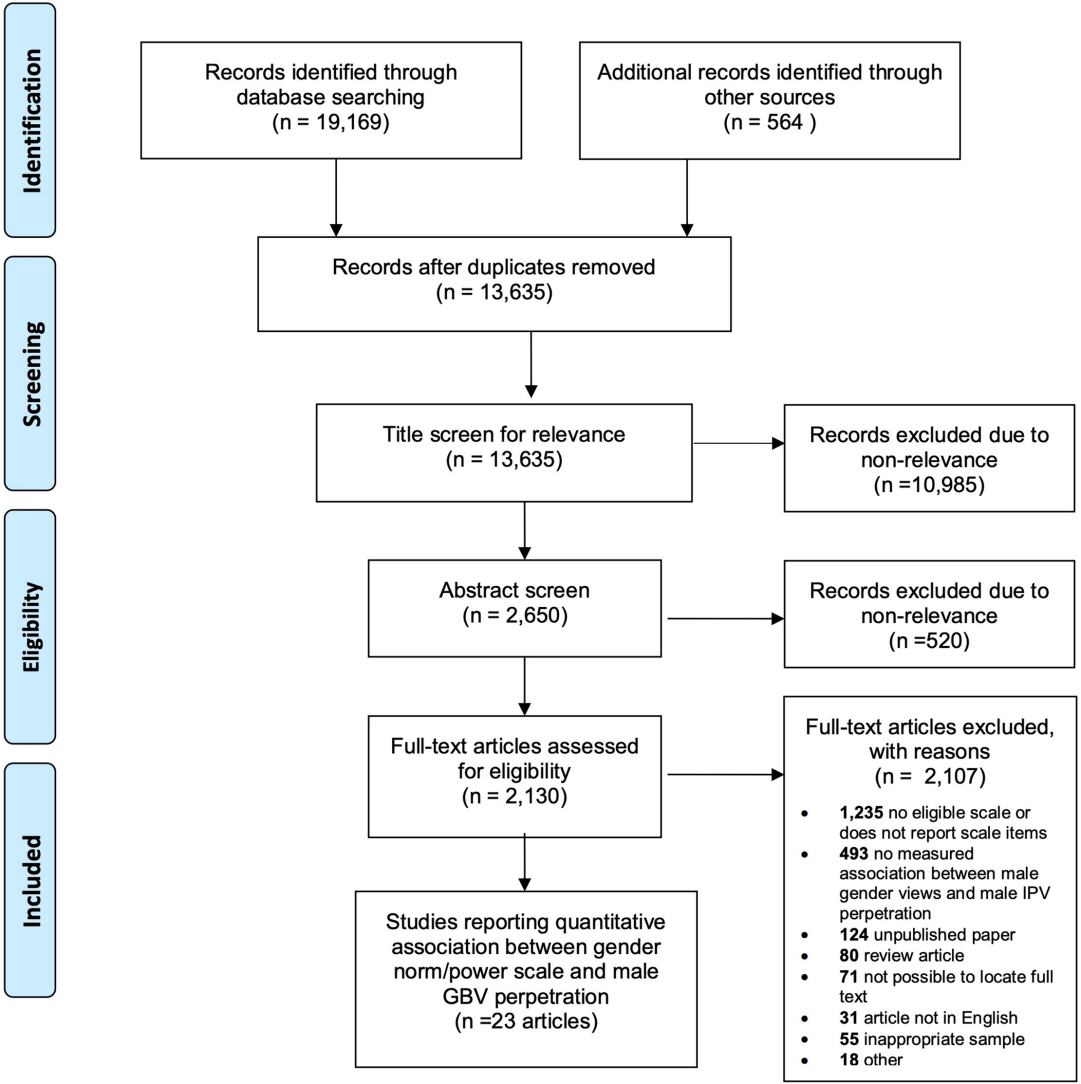
PRISMA flow diagram.

Of 13,635 identified non-duplicate records, 10,985 were excluded following title screen for non-relevance, 520 were excluded at abstract screen for non-relevance and 2,107 were excluded at full-text screen. Reasons for exclusion at the full-text review were most commonly due to lack of an eligible scale or scale items (N = 1,235 excluded) or because no association was reported between male views on gender inequity measures and male IPV perpetration (N = 493 excluded) (see Fig 1).

### Data extraction

We extracted information on the following domains: (1) the sample population (gender, age, geographic location, race/ethnicity), (2) the scale (number of scale items, specific wording used, response options and direction), (3) psychometric properties (reliability/validity information of scale performance) in the study sample, and (4) association with IPV perpetration (analysis method, sample size, effect size and measures of variance, any covariates included in the model). Quantitative associations (irrespective of type of effect coefficient) were extracted for the most adjusted model of male to female perpetration of violence. We extracted information on null associations, even when the full quantitative data were not presented by study authors (e.g., in instances of stepwise model building, where only findings significant at the bivariate level were included in the final model).

### Data synthesis

To inform which types of scales were most sensitive to measuring IPV perpetration, we stratified measures by content of scale items, level of generality (e.g. individual level, such as personal adherence to norms, own behavioral intentions, or feelings or experience of stress related to gender norms and roles, compared to more general levels, such as how married women should behave, or what men generally feel in specific situations, or the respondent’s perceived peer or community acceptance of a given practice), whether the measure was a single-item indicator or a multi-item scale, psychometric properties (e.g., whether the scale internal consistency reliability was acceptable (Cronbach’s alpha ≥0.70)), and scale name. Meta-analysis was not possible given heterogeneity in the types of scales used and their numeric range as well as variation in perpetration outcomes (e.g., type of violence, type of partner, and reporting period). The consistency of scale performance was analyzed by comparing the number of significant findings in the same direction of association for the above-defined gender categories. Scales were also stratified by scale name if the same scale was implemented in multiple studies. A scale measured in multiple settings of the same study was considered to be unique if it was modified for each population.

We synthesized results first by broad thematic areas of scale content, and then by content sub-domains and level of generality. While Tables 1–4 note the specific perpetration outcome measured, our analysis assumed different forms of IPV perpetration (e.g., emotional, physical and sexual) reflect the same underlying construct. For consistency across studies, in our data synthesis we oriented the effect coefficient so the relationship between gender inequity measures and IPV perpetration was in the hypothesized direction (e.g., endorsement of more inequitable norms and gender inequity in divisions of power with greater likelihood of perpetration). In Tables 1–4, a significant finding in this direction is noted as a “positive” association, whereas a significant association between more equitable norms and greater perpetration illustrates a “negative” association.

**Table 1:** Description of studies included in analysis (N = 23).

Reference	Gender inequity measure	Scale category and level of generality^a^	Country	Scale internal consistency reliability^b^	Type(s) of perpetration	Study quality	Indicator summary of significance^c^
Anderson, 2004 [43]	Rules about sex scale	GRV	United States	0.85	Sexual violence	Low	Positive association
Chan, 2011 [47]	Dominance subscale of personal and relationship profile (PRP)	RS CNTRL	China	0.73	Outcome 1: Physical Outcome 2: Sexual Outcome 3: Any violence	High	No association
	Jealousy subscale of personal and relationship profile (PRP)	RS CNTRL		0.87			No association
Das, 2014 [39]	GEM scale (m)	GRV	India	0.70	Outcome 1: Sexual or verbal combined; Outcome 2: Sexual	Medium	Consistently positive
	Condoning violence against girls scale	VAW		0.83			No association
Espinoza, 2012 [31]	Traditionalism subscale from Mirandé sex role inventory (MSRI)	GRV	Mexico	0.85	Outcome 1: Physical Outcome 2: Verbal/ emotional	Medium	Inconsistently negative
Figueredo, 2001 [49]	Self-reported patriarchy scale	GRV	Mexico	0.78	Any spousal abuse	Medium	No association
Fleming, 2015 [17]	GEM scale (m) *for each country*	GRV	Bosnia	0.85	Physical	High	Inconsistently positive
			Brazil	0.89			
			Chile	0.67			
			Croatia	0.83			
			DRC	0.76			
			India	0.75			
			Mexico	0.70			
			Rwanda	0.99			
Fulu, 2013 [3]	GEM scale	GRV	Bangladesh China Cambodia Indonesia Sri Lanka Papua New Guinea	0.72 (overall)	Outcome 1: Physical Outcome 2: Sexual Outcome 3: Physical/sexual Outcome 4: Emotional/ economic	High	Inconsistently positive
	Controlling behavior scale	RS CNTRL		0.61 (overall)			Inconsistently positive
Gage, 2015 [32]	Gender stereotyping scale	GRV	Haiti	0.68	Outcome 1: Psychological Outcome 2: Physical/ sexual	High	No association
	Perceived positive consequences of using DV scale	VAW (incl of peer)		0.74			No association
	DV Acceptance scale	VAW		0.85			No association
	Perceived peer acceptance of DV scale	VAW (peer)		0.88			Consistently positive
Gomez, 2011 [40]	GEM scale	GRV	Brazil	0.82	Psychological, physical or sexual	High	Positive association
Kalichman, 2007 [36]	Hostile attitudes towards women scale (tested as 8 single item indicators)	GRV	South Africa	NR	Sexual	Low	Inconsistent positive
	Male role attitudes scale (tested as 10 single item indicators)	GRV		NR			Mixed effects
	Violence against women scale (tested as five single item indicators)	VAW		NR			Inconsistent positive
Kaura, 2004 [33]	Power satisfaction scale (m)	RS CNTRL	United States	0.76	Emotional, psychological, verbal and physical (combined)	Low	Positive association
Maman, 2010 [46]	Acceptability of violence if woman refuses sex scale	VAW	Tanzania	NR	Physical or sexual	Medium	No association
	Acceptability of violence scale	VAW		0.80			No association
	Male control scale	RS CNTRL		0.83			No association
Nanda, 2014 [44]	GEM scale (m)	GRV	India	0.70	Emotional, economic, physical or sexual (combined)	High	Positive association
Prather, 2012 [34]	Traditional-egalitarian sex roles scale (TESR)	GRV	United States	NR	Romantic aggression	Low	Positive association
Pulerwitz, 2015 [19]	GEM scale	GRV	Ethiopia	0.88	Outcome 1: Physical or sexual Outcome 2: Physical, sexual, emotional (any)	High	No association
Raiford, 2013 [38]	Attitudes towards intimate partner violence scale	VAW	United States	0.80	Physical or sexual	High	No association
Reed, 2011 [37]	Perceptions of peer norms regarding teen dating violence (TDV) perpetration measure	VAW (peer)	United States	NR	Physical, sexual or psychological (combined)	Medium	No association
	Gender attitudes measure	GRV		0.93			Consistently positive
Sambisa, 2010 [41]	Gender role beliefs	GRV	Bangladesh	NR	Outcome 1: Lifetime physical Outcome 2: Past-year physical IPV Outcome 3: Lifetime sexual 4: Any lifetime IPV	Medium	No association
	Attitudes toward IPV scale	VAW		NR			Consistently positive
	Domestic authority scale (household decision-making subscale)	RS CNTRL		NR			No association
	Domestic authority scale (wife’s control of earned cash subscale)	RS CNTRL		NR			No association
Santana, 2006 [50]	Male role attitudes scale (MRAS)	GRV	United States	0.60	Physical or sexual	High	Positive association
Shannon, 2012 [45]	Gender inequity norms scale	GRV	Botswana, Swaziland (Combined)	0.75	Sexual	High	Positive association
Verma, 2008 [23]	GEM scale	GRV	India	0.78	Physical or sexual	Medium	Consistently positive
Verma, 2006 [30]	GEM scale	GRV	India	0.86	Physical	Low	Positive association
Yoshikawa, 2014 [35]	Acceptance of wife beating scale	VAW	Nepal	NR	Outcome 1: Lifetime physical Outcome 2: Past year physical	Medium	Consistently positive

Notes

^a^ Scale category and level of generality: GRV refers to ‘gender role views/norms’, this category is inclusive of individual attitudes, adherence to and expectancies on social roles/norms considered appropriate for men and women; RS CNTRL refers to ‘relationship power/control’, ‘VAW’ refers to acceptance of violence against women. The level of generality refers to the referent group for scale items. Except in cases where ‘peer’ and ‘community’ is specified, the level of generality is the individual respondent–i.e., his personal views, etc.

^b^ Cronbach’s alpha or NR (Not reported).

^c^ For consistency across studies, indicator performance is summarized in the hypothesized direction (i.e., inequitable gender role beliefs, norms or control (with control favoring the male partner) and greater likelihood of IPV perpetration). Inconsistent results noted when direction or level of significance varied by subgroup or outcome (if multiple perpetration outcomes). (m): Modified scale.

**Table 2:** Associations between views on gender roles/norms and IPV perpetration (N = 18 studies).

Citation	Measure^a^ (No. of items)	Indicator attributes		Sample description & size	Scale range^b^	Analysis method	Definition of Violence Perpetration [Male to female]	Results^c^	Indicator summary of significance^d^
**Gender equitable men (GEM) scale**									
Das, 2014 [39]	Modified (m)GEM scale–(15 items)	Gender roles, acceptance of control over women, sexual entitlement, IPV inclusive		Boys ages 10–16 in urban Mumbai, India. Part of school or community-based cricket team (N = 1040)	High vs. low equity; Moderate vs. low equity (**rev**)	Multivariate logistic regression	**Outcome 1:** Perpetrated sexual or verbal violence last 3 months	**Outcome 1:** High v. low equity aOR: 0.29* (0.11, 0.80)	Consistently positive association
								Mod v. low equity aOR: 0.44 (95%CI: 0.18, 1.11)^†^	
							**Outcome 2:** Perpetrated sexual violence last 3 months (incl. harassment)	**Outcome 2:** High v. low equity aOR: 0.09* (0.04, 0.23)	
								Mod v. low equity aOR: 0.31** (0.20, 0.48)	
Gomez, 2011 [42]	GEM scale (24 items)	IPV inclusive, sexual entitlement		Young men ages 15–24 in urban slum of Rio de Janeiro (N = 240)	Mean = 0 (range = -3.1 to 1.5) (**rev**)	Multinomial logistic regression	IPV perpetration in past 6 months (physical, sexual or emotional)	aRRR: 0.69* (0.40, 0.89)	Positive association
Fleming, 2015 [17]	(m)GEM scale–Brazil (11 items)	IPV inclusive		Men ages 18 to 59 surveyed in IMAGES multi-country survey (N = 7806 in pooled sample). Data from Bosnia and Rwanda are nationally representative; other countries are representative of regions/cities surveyed.	Standardized in each country, Mean = 0, SD = 1; score represents respondent’s score relative to other men surveyed in country (**rev**)	Multivariate logistic regression	Physical perpetration (lifetime)	***Brazil*:** aOR: 0.99 (0.79, 1.23)	No association
	(m)GEM scale–Chile (15 items)							***Chile*:** aOR: 0.87 (0.74, 1.01)	No association
	(m)GEM scale–Mexico (11 items)							***Mexico*:** aOR: 0.68** (0.56, 0.82)	Positive association
	(m)GEM scale–Bosnia (15 items)	IPV inclusive						***Bosnia*:** aOR: 0.68** (0.58, 0.80)	Positive association
	(m)GEM scale–Croatia (13 items)	IPV inclusive						***Croatia*:** aOR: 0.87 (0.75, 1.02)	No association
	(m)GEM scale–DRC (13 items)	IPV inclusive						***DRC*:** aOR: 0.92 (0.75, 1.40)	No association
	(m)GEM scale–India (12 items)	IPV inclusive						***India*:** aOR: 1.03 (0.85, 1.24)	No association
	(m)GEM scale–Rwanda (13 items)	IPV inclusive						***Rwanda*:** aOR: 0.94 (0.83, 1.06)	No association
Fulu, 2013 [3]	Gender attitudes scale (10 items) ^e^	IPV inclusive, sexual entitlement		Men ages 18 to 59 surveyed in UN Multi-country study on Men and Violence sampled from a combination of urban and rural sites. Estimates are nationally representative in Cambodia only and regionally representative in Bougainville, Papua New Guinea.	Low equity vs. high or moderate equity	Multinomial logistic regression	**Outcome 1:** Physical IPV perpetration (ever)	***Bangladesh* Outcome 1:** aRR 1.82* (1.35, 2.44)**; Outcome 3:** aRR 2.22*(1.28, 3.84)**; Outcomes 2, 4:** NR (ns)	Inconsistently positive
							**Outcome 2:** Sexual IPV perpetration (ever)	***China* Outcome 1–4:** NR (ns)	
							**Outcome 3:** Physical or sexual IPV perpetration (ever)	***Cambodia* Outcomes 1, 2 and 4:** NR (ns)**; Outcome 3:** aRR 2.31* (1.25, 4.28)	
								***Indonesia* Outcomes 1–4:** NR (ns)	
							**Outcome 4:** Emotional or economic IPV perpetration (ever)	***Sri Lanka* Outcome 1–4:** NR (ns)	
								***Papua New Guinea* Outcomes 1–4:** NR (ns)	
Nanda, 2014 [44]	(m)GEM scale (27 items)	IPV inclusive, sexual entitlement		Men ages 18–49 from 6 states in India (Uttar Pradesh, Rajasthan, Punjab & Haryana, Odisha, Madhya Pradesh, and Maharashtra), representative at each state level (total N = 9205)	Low vs. high/ moderate equity	Multivariate logistic regression	IPV perpetration (emotional, economic, physical or sexual) in past 12 months	aOR: 1.35** (1.15, 1.57)	Positive association
Pulerwitz, 2015 [19]	GEM scale (24 items)	IPV inclusive		Young men ages 15–24 in Ethiopia (N = 729), part of community-engagement intervention	High equity vs. moderate or low (**rev**)	Multivariate logistic regression	Any IPV perpetration (physical, sexual, or emotional)	High-equity GEM scores were associated with a 34% reduction in the odds of perpetration^†^ (95%CI: NR)	No association
Verma, 2008 [23]	(m)GEM scale (15 items)	IPV inclusive		Young men ages 15–29 in Mumbai (urban site) and Gorakhpur (rural site), India (N = 660)	High, moderate, and low equity. Terciles created from continuous score (**rev**)	Multivariate logistic regression	Perpetration of physical or sexual IPV in past 3 months	***Mumbai*** High v. low equity aOR: 0.69* (95%CI: NR)	Consistently positive association
								Mod. v. low aOR: 0.79^†^ (95%CI: NR)	
								***Gorakhpur*** High v. low equity aOR:0.45** (95%CI: NR)	
								Mod. v. low aOR: 0.73* (95%CI: NR)	
Verma, 2006 [30]	GEM scale (24 items)	IPV inclusive		Young men ages 16–24 in Mumbai, India (N = 107)	Continuous (range: NR)	Mean difference	Physical IPV perpetration in past 3 months	NR coefficient*	Positive association
**Other gender norms and belief scales**									
Anderson, 2004 [43]	Rules about sex questionnaire (21 items)		Sexual entitlement	Male students ages 11 to 36 (middle/high school and university) in Indiana, USA (N = 137)	Continuous	Correlation	Frequency of perpetration of sexual coercion	r: 0.30**	Positive association
Espinoza, 2012 [31]	Traditionalism subscale of Mirandé sex role inventory (MSRI) (17 items)			Young men age 15–18 in high school in Monterrey, Mexico (N = 75)	Continuous	Multiple linear regression	**Outcome 1:** physical IPV **Outcome 2:** emotional IPV	**Outcome 1** Adj.B: -0.44** SE: NR	Inconsistently negative association
								**Outcome 2** Adj. B: -0.03, SE: NR	
Figueredo, 2001 [49]	Patriarchy scale (11 items)		IPV inclusive, male control over wealth	Men in Sonora, Mexico who were in a committed relationship during past year. Mean age = 33 (N = 106)	Continuous	Multiple linear regression	IPV perpetration (any type)	Adj Beta: -0.06, SE: NR	No association
Gage, 2016 [32]	Gender stereotyping scale (7 items)			Male high school students in Port-au-Prince who had ever been on a date (N = 342)	Continuous	Multiple linear regression	**Outcome 1:** Psychological IPV perpetration **Outcome 2:** Physical/ sexual IPV perpetration (ever)	**Outcome 1** Adj. B: 0.27, SE: 0.12	No association
								**Outcome 2** Adj B: 0.23, SE: 0.20	
Kalichman, 2007 [36]	*Male role attitudes scale items [tested individually*]			Men older than 18 in Cape Town, South Africa (N = 435)	NR	Multivariate logistic regression	Sexual assault perpetration (ever)		
	It is essential for a man to get respect from others							aOR: 0.70 (0.40, 1.40)	No association
	A man always deserves the respect of wife & children							aOR: 0.50* (0.20, 0.90)	Negative association
	I admire a man who is very confident							aOR: 0.50* (0.20, 0.80)	Negative association
	A man will lose respect if he talks about his problems							aOR: 0.80 (0.50, 1.20)	No association
	A young man should be physically tough, even if he is not big							aOR: 0.70 (0.40, 1.30)	No association
	I don’t think a husband should have to do housework							aOR: 1.60** (1.10, 0.60)	Positive association
	Men are always ready for sex							aOR: 0.90 (0.50, 1.40)	No association
	A man who does not provide for his family is less than a man							aOR: 1.10 (0.60, 1.90)	No association
	Hostile attitudes towards women scale items [tested individually]			Men older than 18 in Cape Town, South Africa (N = 435)	NR	Multivariate logistic regression	Sexual assault perpetration (ever)		
	Many women seek special favors that place them over men							aOR: 1.70* (1.10, 2.90)	Positive association
	Most women think innocent remarks or acts are meant to hurt them							aOR: 1.10 (0.70, 1.80)	No association
	Women are too easily offended							aOR: 1.30 (0.80, 2.30)	No association
	Most women fail to appreciate all that men do for them							aOR: 1.10 (0.70, 1.90)	No association
	Women who have jobs and make money should give the money to their man to pay bills		Male control over wealth					aOR: 1.20 (0.70, 1.90)	No association
	Women only work so they can gain power and control over men		Male control over wealth					aOR: 1.70* (1.10, 2.70)	Positive association
	Once a woman makes money she usually tries to control her man		Male control over wealth					aOR: 1.40 (0.90, 2.20)	No association
	It is difficult for a man to work at a job where a woman is the boss							aOR: 0.80 (0.50, 1.20)	No association
	A woman should only show her man respect in front of other people.							aOR: 0.80 (0.50, 1.30)	No association
	Some women need a man to help them survive							aOR: 2.20** (1.20, 4.10)	Positive association
Reed, 2011[37]	Gender attitudes scale (13 items)		Sexual entitlement	Young men ages 14–20, seeking healthcare at clinics in Boston, USA (N = 320)	Continuous	Multiple linear regression	Teen dating violence perpetration (physical, sexual or emotional) (ever)	**Total sample** Adj. beta: 1.50*, SE: 0.60	Consistently positive
								**Sexually active subgroup** Adj. beta: 2.00*, SE: 0.90	
Sambisa, 2010 [41]	Attitudes about wife working outside the home (2 items)		Male control over wealth	Married men ages 15 to 49 in Bangladesh (N = 8320)	Support for wife working outside home in at least once instance (vs. none)	Multivariate logistic regression	**Outcome 1**: Lifetime physical IPV perpetration	**Outcome 1** aOR: 0.92, (95%CI: NR)	No association
							**Outcome** 2: Past-year physical IPV perpetration	**Outcome 2** aOR: 0.87, (95%CI: NR)	
							**Outcome 3:** Lifetime sexual IPV perpetration	**Outcome 3** aOR: 1.01, (95%CI: NR)	
							**Outcome 4:** Lifetime IPV perpetration	**Outcome 4** aOR: 0.90, (95%CI: NR)	
Santana, 2006 [50]	Male role attitudes scale (8 items)			Men ages 18–35 who are sexually active in the past 3 months, English and/or Spanish and receive services at clinics in Boston, USA (N = 283)	Continuous	Multivariate logistic regression	Physical or sexual IPV perpetration in the past year	aOR: 1.80* (1.10, 2.09)	Positive association
Shannon, 2012 [45]	Gender inequity norms scale (6 items)		IPV inclusive, sexual entitlement	Men ages 23 to 36 in Botswana and Swaziland (N = 999)	NR	Multivariate logistic regression	Rape perpetration	aOR: 2.19*, (1.22, 3.51)	Positive association
Prather, 2012 [34]	Traditional-egalitarian sex roles scale (TESR) (20 item)		Male control over wealth	College students ages 18–25 in USA (N = 260; 77 men, 183 women, finding adjusts for gender)	Continuous	Multiple linear regression	Psychological IPV perpetration	Std Adj. beta: 0.25** (95%CI: NR); Respondent sex did not moderate relationship between sex role attitudes and perpetration (Std adj. beta:0.08)	Positive association

Notes: NR indicates not reported.

^a^ This category is inclusive individual beliefs, attitudes and expectancies on social norms and roles considered appropriate for men and women

^b^Scales are coded so that higher score represents less equitable beliefs, **(rev)**: indicates reverse orientation of indicator response scale (higher score signifies more equitable views)

^c^We report outcomes for the most adjusted or final statistical model using the following terminology: aOR = adjusted odds ratio; aRR = adjusted risk ratio; Adj beta = adjusted beta coefficient, Std Adj beta = standardized adjusted beta coefficient, r = correlation coefficient (unadjusted). Unless indicated the variance measure reported is the 95% confidence interval.

^d^ For consistency across studies, indicator performance is summarized in the hypothesized direction (e.g., less equitable beliefs and greater likelihood of IPV perpetration), Inconsistent results noted when direction or level of significance varied by subgroup or outcome (if multiple reported)

^e^Constructed from GEM scale and Medical Research Council men’s health and relationship study.

*p<0.05

**p<0.001

† Marginal significance at p<0.10

**Table 3:** Associations between measures of acceptance of violence against women and IPV perpetration (N = 9 studies).

Citation	Measure (No. of items)	Indicator attributes	Sample description & size	Scale range^a^	Analysis method	Definition of Violence Perpetration [Male to female]	Adjusted results^b^	Indicator summary of significance^c^
Das, 2014 [39]	Condoning violence against girls (9 items)	Specific justification	Boys ages 10–16 in urban Mumbai, India. Part of school or community-based cricket team (N = 1040)	High vs. low equity; Moderate vs. low equity (**rev**)	Multivariate logistic regression	**Outcome 1:** Perpetrated sexual or verbal violence in last three months	**Outcome 1:** NR (ns)	No association
						**Outcome 2:** Perpetrated sexual violence in last three months (incl. harassment)	**Outcome 2:** NR (ns)	
Fleming, 2015 [17]	Attitudes towards violence against women (1 item)	General acceptance	Men ages 18 to 59 surveyed in IMAGES multi-country survey (N = 7806 in pooled sample). Data from Bosnia and Rwanda are nationally representative; other countries are representative of regions/cities surveyed.	Standardized in each country, Mean = 0, SD = 1; score represents respondent’s score relative to other men surveyed in country (**rev**)	Multivariate logistic regression	Physical perpetration (lifetime)		Inconsistently positive association
							**Chile** aOR: 1.90** (1.18, 3.04)	
							**Mexico** aOR: 2.55** (1.31, 4.97)	
							**Bosnia** aOR: 1.34 (0.93, 1.95)	
							**Brazil** aOR: 1.92** (1.18, 3.12)	
							**Croatia** aOR: 3.14** (2.00, 4.95)	
							**DRC** aOR 1.52 (0.99, 2.34)	
							**India** aOR: 1.28 (0.89, 1.85)	
							**Rwanda** aOR: 1.33 (0.99, 1.80)	
Gage, 2016 [32]	Perceived peer acceptance of domestic violence (8 items)	Specific justification, peer norms	Male high school students in Port-au-Prince who had ever been on a date (N = 342)	Continuous	Multiple linear regression	**Outcome 1:** Psychological IPV perpetration **Outcome 2:** Physical/ sexual IPV perpetration (ever)	**Outcome 1:** Adj. beta: 0.47**, SE: 0.12	Consistently positive association
							**Outcome 2:** Adj. beta: 0.55** SE: 0.20	
	Domestic violence acceptance (8 items)	Inclusive of specific justification		Continuous			**Outcome 1:** Adj. beta: -0.04, SE: 0.14	No association
							**Outcome 2:** Adj. beta: 0.03, SE: 0.23	
	Perceived positive consequences of using domestic violence (3 items)	General acceptance, inclusive of peer norms		Continuous			**Outcome 1:** Adj. beta: 0.47, SE: 0.28	No association
							**Outcome 2**: Adj. beta: 0.41, SE: 0.46	
Kalichman, 2007 [36]	*Acceptance of violence against women scale items [tested individually]*		Men older than 18 in Cape Town, South Africa (N = 435)	NR	Multivariate logistic regression	Sexual assault perpetration (ever)		Inconsistently positive association
	A woman who talks disrespectful to a man in public should expect trouble	Specific justification					aOR: 2.70** (1.4, 4.9)	
	Hitting a woman is sometimes necessary to keep her in line	Specific justification					aOR: 2.90** (1.8, 4.7)	
	It is understandable that a man will hit his women if she is disrespectful of him	Specific justification					aOR: 2.20** (1.30, 3.40)	
	There are times when a man should hit his woman because of things she has done	General acceptance					aOR: 2.20** (1.40, 3.60)	
	A man is expected to discipline his woman	General acceptance					aOR: 1.20 (0.70, 1.90)	
Maman, 2010 [46]	Acceptability of violence scale (9 items)	Specific justifications; inclusive of sexual entitlement	Young men ages 16–24 who were sexually active, Dar Salaam, Tanzania (N = 360)	It is always unacceptable for a woman to refuse sex vs. it is acceptable in at least one of 9 conditions	Multivariate logistic regression	IPV perpetration (at least one physical or sexual violent act with partner)	**Violence is always unacceptable v. no conditions** aOR: 1.63 (0.54, 4.93)	No association
							**Violence is acceptable in some v. no conditions** aOR: 1.60 (0.82, 3.12)	
	Acceptability of violence if woman refuses sex scale (4 items)	Specific justifications; inclusive of sexual entitlement		It is always unacceptable for a woman to refuse sex vs. it is acceptable in at least one of four sexual scenarios	Multivariate logistic regression	IPV perpetration (at least one physical or sexual violent act with partner)	aOR: 0.79 (0.37, 1.68)	No association
Raiford, 2013 [38]	Attitudes towards intimate partner violence scale (12 items)	Specific justification and general acceptance	African American men who were single, heterosexual and had unprotected sex in the past 30 days in Atlanta, USA (N = 65)	Continuous	Multiple linear regression	IPV perpetration (physical or sexual) past 3 months	Adj. beta: 0.07, SE: NR	No association
Reed, 2011[37]	Perceptions of peer norms regarding teen dating violence (TDV) perpetration measure (2 items)	Specific justification; inclusive of sexual entitlement; Peer norms	Young men ages 14–20, seeking healthcare at clinics in Boston, USA (N = 320). Includes men both sexually active and non	NR	Multivariate logistic regression	Teen dating violence perpetration (physical, sexual or emotional) (ever)	**Total sample:** aOR: 1.50 (0.80, 3.10)	No association
							**Sexually active sample:** aOR: 2.80 (0.90, 9.30)	
Sambisa, 2010 [41]	Attitudes toward IPV scale (5 items)	Specific justification	Married men ages 15 to 49 in Bangladesh (N = 8320)	NR	Multivariate logistic regression	**Outcome 1**: Lifetime physical IPV perpetration	**Outcome 1:** aOR: 2.02** **(**95%CI: NR)	Consistently positive association
						**Outcome** 2: Past-year physical IPV perpetration	**Outcome 2:** aOR: 1.95** **(**95%CI: NR)	
						**Outcome 3:** Lifetime sexual IPV perpetration	**Outcome 3:** aOR:1.57** (95%CI: NR)	
						**Outcome 4:** Any lifetime IPV perpetration	**Outcome 4:** aOR: 2.17* (95%CI: NR)	
Yoshikawa, 2014 [35]	Husband’s acceptance of wife beating scale (6 items)	Specific justification; inclusive of sexual entitlement	Married couples ages 18 to 49 in Nepal (N = 717)^d^	1 = at least one affirmative response, 0 = no affirmative responses	Multivariate logistic regression	**Outcome 1:** Lifetime physical IPV perpetration	**Outcome 1:** aOR: 2.58** (1.36, 4.91)	Consistently positive association
						**Outcome 2:** Past year physical IPV perpetration	**Outcome 2:** aOR: 2.78** (1.41, 5.51)	

Notes: NR indicates not reported.

^a^ Scales are coded so that higher score represents greater justification of violence against women.

^b^ We report outcomes for the most adjusted or final statistical model using the following terminology: aOR = adjusted odds ratio; Adj beta = adjusted beta coefficient, exp(b) = log odds coefficient. Unless otherwise indicated, the variance measure is 95% confidence interval.

^c^ For consistency across studies, indicator performance is summarized in the hypothesized direction (e.g., greater endorsement of violence against women and greater likelihood of IPV perpetration). Inconsistent results noted when direction or level of significance varied by subgroup or outcome (if multiple reported).

^d^ Models male perpetration of IPV controlling for husband and wife specific factors.

*p<0.05

**p<0.001

† Marginal significance at p<0.10

**Table 4:** Associations between measures of relationship power and control and IPV perpetration (N = 5 studies).

Citation	Measure (No. of items)	Indicator attributes	Sample description & size	Scale range^a^	Analysis method	Definition of Violence Perpetration [Male to female]	Adjusted results^b^	Indicator summary of significance^c^
Chan, 2011 [47]	Dominance subscale of Personal and Relationship Profile (PRP) (9 items)	Male authority, disparagement of partner, restrictiveness of partner	Adult married men ages 16 and older in Hong Kong, China (N = 2225)	Continuous	Multivariate logistic regression	**Outcome 1:** Physical IPV perpetration **Outcome 2:** Sexual IPV perpetration **Outcome 3:** Any violence or injury perpetration	**Outcome 1:** aOR: 0.61 (0.18, 2.06)	No association
							**Outcome 2:** aOR: 2.16 (0.51, 9.12)	
							**Outcome 3:** aOR: 0.87 (0.32, 2.40)	
	Jealousy subscale of Personal and Relationship Profile (PRP) (8 items)	Anticipated emotional response		Continuous			**Outcome 1:** aOR: 0.93 (0.54, 1.59)	No association
							**Outcome 2:** aOR: 0.71 (0.36, 1.42)	
							**Outcome 3**: aOR: 0.88 (0.56, 1.40)	
Fulu, 2013 [3]	Controlling behavior scale (8 items)	Inclusive of sexual behavior	Men ages 18 to 59 surveyed in UN Multi-country study on Men and Violence sampled from a combination of urban and rural sites. Estimates are nationally representative in Cambodia only and regionally representative in Bougainville, Papua New Guinea.	Low equity vs. high or moderate equity. Terciles created from continuous score.	Multinomial logistic regression	**Outcome 1:** Physical IPV perpetration (ever)	***Bangladesh***	Inconsistently positive association
							**Outcome 1:** aOR: 2.27* (1.10, 4.67)	
							**Outcome 3:** aOR 4.10* (1.72, 9.73)	
							**Outcomes 2, 4:** NR (ns)	
						**Outcome 2:** Sexual IPV perpetration (ever)	***China***	
							**Outcome 2**: aOR: 3.40* (1.39, 8.30)	
							**Outcomes 1, 3, 4:** NR (ns)	
						**Outcome 3:** Physical or sexual IPV perpetration (ever)	***Cambodia***	
							**Outcome 2**: aOR: 2.55* (1.30, 4.98)	
							**Outcomes 1, 3, 4:** NR (ns)	
						**Outcome 4:** Emotional or economic IPV perpetration (ever)	***Indonesia***	
							**Outcome 2**: aOR: 2.50* (1.14, 5.49)	
							**Outcomes 1, 3, 4:** NR (ns)	
							***Sri Lanka***	
							**Outcome 1:** aOR: 3.30* (1.61, 6.75)	
							**Outcome 4:** aOR: 5.13* (1.79, 14.66)	
							**Outcomes 2–3:** NR (ns)	
							***Papua New Guinea*** **Outcomes 1–4:** NR (ns)	
Kaura, 2004 [33]	Modified power satisfaction scale (6 items)	General relationship decisions	Male university students, USA (N = 352)	Continuous	Multiple linear regression	Frequency of IPV perpetration (emotional, psychological, verbal, and physical)	Adj. beta: 0.19**, SE: NR	Positive association
Maman, 2010 [46]	Male control scale (3 items)	Male autonomy, partner control	Young men ages 16–24 who were sexually active, Dar Salaam, Tanzania (N = 360)	Always unacceptable for a woman to refuse sex vs. acceptable in at least 1 of 4 conditions	Multivariate logistic regression	IPV perpetration (at least one physical or sexual violent act with partner)	**Violence is always unacceptable v. no conditions** aOR: 1.31, (0.30, 5.83)	No association
							**Violence is acceptable in some v. no conditions** aOR: 1.42, (0.37, 5.50)	
Sambisa, 2010 [41]	Domestic authority scale (6 items)	Household decision-making; women’s mobility	Married men ages 15 to 49 in Bangladesh (N = 8320)	Dichotomized: High/ moderate vs. low control)	Multivariate logistic regression	**Outcome 1**: Lifetime physical IPV perpetration **Outcome 2**: Past-year physical IPV perpetration **Outcome 3:** Lifetime sexual IPV perpetration **Outcome 4:** Any lifetime IPV perpetration	**Outcome 1:** aOR: 1.04, (95% CI: NR)	No association
							**Outcome 2:** aOR: 1.06, (95% CI: NR)	
							**Outcome 3**: aOR: 1.18, (95% CI: NR)	
							**Outcome 4:** aOR: 1.12, (95% CI: NR)	
	Wife’s control of cash she earned (2 items)	Male control over wealth		Husband controls wife’s cash vs. egalitarian			**Outcome 1** aOR: 1.01, (95%CI: NR)	No association
							**Outcome 2** aOR: 0.96, (95%CI: NR)	
							**Outcome 3** aOR: 1.60, (95%CI: NR)	
							**Outcome 4** aOR: 1.07, (95%CI: NR)	

Notes: NR indicates not reported.

^a^ Scales are coded so that higher score represents greater male power/ control in relationship.

^b^ We report outcomes for the most adjusted or final statistical model using the following terminology: aOR = adjusted odds ratio; Adj beta = adjusted beta coefficient, exp(b) = log odds coefficient. Unless otherwise indicated, the variance measure is the 95% confidence interval.

^c^ For consistency across studies, indicator performance is summarized in the hypothesized direction (e.g., higher male control and greater likelihood of IPV perpetration). Inconsistent results noted when direction or level of significance varied by subgroup or outcome (if multiple reported).

*p<0.05

**p<0.001

† Marginal significance at p<0.10

### Quality assessment rating

The risk of bias in individual studies was assessed using a modified quality appraisal checklist for quantitative studies [29]. Criteria included study design (e.g., cross-sectional, observational cohort, quasi-experimental or experimental design), representativeness of the source population in the study sample, ascertainment of the exposure (e.g., whether the masculinity measure was clearly defined, whether validity or reliability data were presented for the gender measure), assessment of the outcome (e.g., whether the violence outcome was well-defined, the reporting period was reasonable, whether types of IPV were disaggregated or combined), the potential that confounding factors were identified and controlled for, whether analytical methods were appropriate, whether the precision of the estimate was provided or appropriate, among other criteria. For each checklist criteria, studies could receive between 0 to 2 points, with a possible range of 0 to 22. We considered studies with total scores between 0 to 11 points as low quality, 12 to 15 as medium quality and 16 or higher as high quality.

## Results

### Characteristics of included studies and measures

We identified 23 studies that measured the association between a measure of gender inequity and male perpetration of IPV (Fig 1). Nearly all (20) studies were observational and used a cross-sectional design. Three studies were quasi-experimental and examined the effect of intervention activities on support for inequitable gender norms and partner violence, among other outcomes [19,23,30]. One study assessed change in gender views on the likelihood of IPV perpetration over time [19], while the remaining associations relevant to this review were cross-sectional (e.g., cross-sectional comparison at baseline or endline). Within this sample of studies, ten studies were considered high quality, eight medium, and five low quality (Table 1). Five studies were implemented among combined samples of males and females but disaggregated findings by respondent sex [31–35]. In these cases, the male coefficient is presented.

More than one-third of studies took place in Asia or the Pacific, while about 20% were located in North America, Latin America and Sub-Saharan Africa, respectively. By country, most studies took place in the United States (6 studies), followed by India (5). In total, data were extracted for 64 measures in relation to IPV perpetration. The majority of measures (39 of 64) were multi-item scales, while 25 were single item indicators. A higher proportion of multi-item scales were positively associated with IPV perpetration (54%) relative to single-item scales (36%). Of multi-item scales, the majority (69%, N = 27) reported some indicator of scale internal consistency reliability that was considered acceptable (e.g., Cronbach’s alpha ≥0.70), 10% (N = 4) reported a Cronbach’s alpha of <0.70, and 20% (N = 8) of multi-item scales did not report any measure of reliability among the sample population. A slightly higher proportion of multi-item scales with moderate or higher internal consistency reliability (48%, or 13 of 27 scales) were positively associated with IPV perpetration relative to multi-item scales with no data reported (3 of 8, 37%).

### Overall association between gender inequity measures and IPV perpetration

In total, 64 measures (i.e., multi-item scales or single item indicators) of gender norms, views, relations, and practices were identified. The high number of measures is partially due to the disaggregation of two scales into multiple single item indicators (Hostile Attitudes towards Women scale and Male Role Attitude scale) in one low quality study, rather than reporting the association with the overall scale [36]. Additionally, one high-quality multi-country study implemented the GEM scale, with a modified (i.e., unique) version tailored for each setting [17]. Overall, about half of measures (N = 35, 55%) were not associated with male perpetration of IPV. Greater perceived gender inequitable norms, individual endorsement of traditional gender norms or violence against women, or more male power/control in relationships were positively associated with male perpetration of IPV in 29 of the 64 identified measures (45%). A negative (or inverse) association was documented in three instances in two studies [31,36]. If we look at results at the level of the study, most (74%, 17 out of 23) found at least one positive relationship between a gender inequity measure and an IPV outcome.

### Specific scale subtypes

While the majority of examined associations, did not find positive correlations with IPV perpetration, this finding varied by the type of construct measured. We empirically categorized three broad scale types: views on gender roles/norms, acceptance of violence against women, and relationship power and control. Within each category, we further note the level of generality/ reference group (e.g., individual-level view, or perception of peer or community level norms) and common sub-domains of question content (Tables 1–4). We note that all analyses were at the level of the individual (e.g., individual’s perceived peer or community acceptance of a given practice).

A total of 42 measures (18 studies) reflected views on gender roles or norms, the largest category of identified scales. Views on gender roles/norms encompassed individual-level attitudes and personal adherence to gender norms (henceforth collectively referred to as “views”). Example scale items include “*A man should have the final word about decisions in his home*” [17], or “*I admire a boy/man who is totally sure of himself*” [37]. Measures of gender views encompassed multiple sub-domains of specific content areas, such as male beliefs of sexual entitlement, control over wealth, and the acceptability of use of violence against women, either as a demonstration of masculinity or to enforce traditionally defined gender roles for girls and women. The second largest category of identified measures (20 measures) exclusively reflected views on the acceptance of violence against women (11 studies), either in general (e.g., “*some women deserve to be slapped*” [38] or in specific scenarios (e.g., “*when she replies back when harassed by boys*” [39]. Finally, seven measures represented power and control dynamics in relationships (5 studies). Relationship power and control measures included both self-reported male behaviors to limit the autonomy and decision-making of their partners, or men’s anticipated controlling reaction (either behavioral or emotional) towards their partner’s actions. We discuss variation in the consistency of observed findings by these broad scale categories in the sections that follow. We also synthesize findings by above-noted sub-domains of content area and level of generality (Tables 2–4).

### Measures assessing views on gender roles / norms

Eighteen (18) studies (eight high quality, six moderate and four low quality) tested 42 measures of views on gender roles/norms and IPV perpetration (Table 2). No gender norm measures were identified, i.e., measures that reflected the respondent’s perception of what a reference group (e.g., peers or community) does or approves of in terms of socially accepted roles and behaviors of men or women. Further, no study aggregated individual-level views to approximate a community norm. Across all 42 measures, greater endorsement of gender inequitable views was positively correlated with IPV perpetration in 17 of 42 (40%) measures. About half of measures (22) found no significant association with perpetration, and in a minority (3), an inverse association was documented [31,36]. Of note is about half (12 of 22) of the measures with no significant correlation with perpetration and two of the three items with an observed negative association with violence perpetration were single-item indicators as opposed to multi-item scales. When only multi-item scales are considered, 13 out of 24 (54%) were significantly positively correlated with violence perpetration. When examined at the level of study, out of 18 papers that assessed measures of views on gender roles/norms, 13 (72%) found at least one positive association between such a measure and IPV perpetration. This finding did not substantially change considering only moderate and high-quality studies.

#### GEM scale

The most commonly used scale in this broad category was the GEM scale, which asks about participant endorsement of gender roles/norms. Fifteen scales were derived from eight studies (five high quality, two moderate and one low-quality). One high-quality study implemented the GEM scale in eight countries, adapted for each setting [17]. Example items of the GEM scale include, “*A woman’s most important role is to take care of her home and cook for her family” and*, *“To be a man*, *you need to be tough”*. In the great majority of studies—seven out of the eight studies—the GEM scale was positively associated with perpetration of IPV in at least one instance. When examined at the level of the scale (considering each modified version of the GEM scale as unique), the GEM scale was positively associated with at least one form of IPV perpetration in eight out of the 15 (53%) measures. We also note some trends by geographic area. The GEM scale was most often implemented in India, and inequitable beliefs were positively associated with male IPV perpetration in four of five Indian settings [17,23,30,39]. The GEM scale was also implemented more than once in Brazil, with inconsistently positive findings [17,40]. Considering the scales that only implemented the unmodified GEM scale, two out of three studies documented a significant positive association with IPV perpetration (Table 2).

#### Gender views inclusive of attitudes towards violence against women

Measures of views on gender roles/norms often included questions about participant’s attitudes towards or acceptance of use of violence against women (15 scales), (e.g., “*A woman should tolerate violence to keep her family together*”) [17], in addition to questions about participant’s views about other gender norms. The 15 scales were derived from 10 studies (six high quality, three moderate and one low quality). One high quality study implemented eight modified versions of the GEM scale, six which were inclusive of views on the acceptance of violence against women [17]. Overall, the majority of scales (8 of 15, 53%) inclusive of justification for violence were positively associated with perpetration of violence. In contrast, a lower proportion of the scales which did not include attitudes towards violence, (9 of 27, 33%) were associated with perpetration. No scales inclusive of views on violence demonstrated a negative association with perpetration.

#### Gender views on male control over wealth

Male authority or control regarding finances (e.g., “*If the husband is making enough money*, *do you believe it is acceptable for women to work outside the home*” [41], was another common component of scales assessing participants views on gender roles/norms, reflected in five measures (3 studies) [34,36,41]. In two out of the five measures, a positive association was documented between the scale and male IPV perpetration. However, two of the three studies were low quality; one was moderate quality. The small number of studies and overall quality limits the ability to draw stronger conclusions between views on male control over wealth and IPV perpetration.

#### Gender views regarding male sexual entitlement

Seven gender view scales included questions about male sexual entitlement to women [3,37,39,42–45]. Male sexual entitlement included male beliefs about conditions where sex was expected from women, or agreement that men should be sexually aggressive (e.g., “*A man has the right to have sex with his wife/partner when he wants”* or promiscuous, “*A man needs other women*, *even if things with his wife are fine”* [42]. All seven studies (four high, two moderate and one low quality) documented a positive association with IPV perpetration, although in one of the studies the association was inconsistently positive. This high quality, multi-country study found a positive association among men in Bangladesh and Cambodia, but not in four other Asian countries [3]. Notably, only one gender role scale, the Rules About Sex scale, focused exclusively on male beliefs regarding conditions where women were expected to give in to sex [43]. This study documented a positive association with IPV, but was low quality.

### Acceptance of violence against women

Nine studies considered 16 measures regarding acceptance of violence against women (Table 3). Of these, four were considered high quality [17,32,38,39], four medium quality [35,37,41,46], and one low quality [36]. Roughly half of measures (9 of 16, 56%) were positively correlated with male perpetration of IPV in at least one instance. The same was true when we looked at the level of study–about half the studies (5 out of 9) found at least one positive association between a scale measuring endorsement of violence against women and IPV perpetration. All positive associations were documented in relation to physical or sexual violence perpetration and most studies (four of five) were moderate or high quality. Emotional violence was measured in one high-quality study, and no association with acceptance of IPV was observed [32]. One high quality, multi-country study which examined acceptance of violence against women in eight low and middle-income countries (LMIC) found the positive association with physical IPV also varied by setting (a significant positive association was observed in 4 of 8 countries) [17].

Measures of IPV acceptance were most often comprised of situation-specific justifications for using violence (12 of 16 measures). Typically, the justifications related to use of violence as a means to enforce socially proscribed gender roles and responsibilities, such as “*A husband is justified for beating his wife if she fails to provide food on time*” [41]. Less often justifications related to use of violence as a means to express male love or commitment. Four measures reflected general acceptance of IPV, irrespective of the context in which it occurred [17,32,36]. Two measures of general acceptance (from one high and one low quality study) were positively associated with perpetration, although one varied by country setting [17,36]. Similarly, half of measures which included situation-specific justifications (6 of 12 measures) were associated with perpetration in at least one instance.

While most measures of acceptance of violence reflected individual-level views (13 of 16), three measures (two from a high quality study, one from a moderate quality study) reflected perceived peer acceptance of IPV or the frequency of peer IPV perpetration [32,37]. One of these scales included peer norms in addition to questions about the respondent’s own views of domestic violence [32]. No measures reflected normative beliefs regarding community acceptance of gender-based violence. Only one of the two norm scales, perceived peer acceptance of domestic violence, was positively associated with perpetration [32]. The measure that included, but did not exclusively measure peer norms, was not associated with perpetration. Taken together, these results raise the question whether individual-level endorsement of norms is more salient to IPV perpetration than perceived peer norms, but the number of studies assessing norms was too small to make conclusions in this regard.

### Relationship power and control

Five studies (two high quality studies, two moderate quality and one low quality) included seven distinct measures of power and control in relationships (Table 4) [3,33,41,46,47]. Most measures reflected behavioral practices, however anticipated emotional reactions (e.g., jealousy) towards a partner’s actions were also included in the scales, (e.g., “*I would be upset if someone hugged my partner a little too long”)* [47]. Of these seven power and control measures, two (from one high and one low quality study) were positively associated with violence perpetration in at least one instance [3,33]. The high-quality study assessed men’s controlling behaviors in relation to perpetration of physical, sexual, physical/sexual combined or emotional/economic violence, respectively, in six countries [3]. The scale demonstrated an inconsistently positive association depending on the setting and type of violence. In four of the six settings, relationship power and control was positively associated with physical violence, in three settings it was associated with sexual violence, and in one setting it was associated with emotional/economic violence. This particular scale was the only relationship power/control measure to reference sexual behavior (among other aspects of partner control in the relationship), although these items reflected behavioral and emotional expectations rather than explicit behaviors (e.g., “*When I want sex*, *I expect my partner to agree*”, “*If my partner asked me to use a condom*, *I would get angry”*). The other study which documented a positive association (low quality) assessed a measure of relationship decision-making dominance, and the respondent's satisfaction with the relative distribution of power. The study found that greater dissatisfaction with relationship power among men was associated with greater likelihood of any type of IPV perpetration [33]. The five remaining measures of power and control showed no association with violence perpetration.

## Discussion

This systematic review finds that overall, over half of gender inequity measures–i.e., those that measured gender norms, views on gender roles/ norms, endorsement of violence against women and gender-related inequities in relationship power and control—were not associated with male perpetration of IPV. This suggests that if, in fact gender inequities play a salient role in IPV perpetration as hypothesized, there may be considerable scope for improving our scales and indicators for men in these domains. Indeed, we found substantial variation by the scale content category and by specific scales used. Overall, of the three broad categories of measures considered, measures that included acceptance of violence against women were most often associated with male perpetration of IPV (56%), followed by views on gender roles / norms (40%) and lastly by measures of relationship power and control (29%). We also find that subsets of these categories–such as scales which encompassed male sexual entitlement to women–which were associated with IPV in seven out of seven studies–or the GEM scale—which was associated with IPV at least once in seven out of eight studies—tended to be more consistently positively associated with perpetration.

Male control over women is one way men demonstrate and enforce their masculine identity [17]. One might hypothesize that controlling behavior may be more proximal to violence perpetration than endorsement of norms as it suggests that such views are already being acted upon. It was thus somewhat surprising that measures of gender inequities in relationship power and control were the category of scales least consistently associated with IPV perpetration. While this is consistent with the findings of the UN study–which found that men’s reports of gender inequitable attitudes explained a greater proportion of IPV perpetration than their reports of controlling behaviors [3]–there are a number of possible explanations for our finding. In most instances the content of control scales identified in this review reflected more moderate and general behaviors (e.g., “*I generally have the final say when my partner and I disagree”*) as opposed to specific restrictive controlling behaviors. Indeed, only one identified relationship control scale specifically included control over sexual behavior [3]. This study was one of the two relationship control measures which documented a positive association with IPV perpetration. The less explicit and more general content of the control measures may explain the weaker overall association observed between the control measures and IPV perpetration. Moreover, we note that because the IPV perpetration studies in this review were conducted among males, scales more widely used among females to measure equality in relationships, such as the Sexual Relationship Power Scale did not appear in the final sample of studies we analyzed here. It may be that these scales would have different results.

Scales most sensitive to measuring males’ self-report of IPV perpetration tended to be more explicit about views towards male sexual entitlement. That is, views on gender role / norm measures inclusive of views on violence against women or which reflected male sexual entitlement to women were more consistently associated with IPV perpetration than measures that did not include these content areas. Notably, none of the measures of gender inequities in relationship control, the category of scales least consistently associated with IPV perpetration, referenced violence. Scales that reflected other gendered behavior domains, such as male control over household wealth, were less consistently associated with IPV perpetration. These results suggest that the antecedents—such as attitudes, anticipated reactions, norms, etc.—most strongly correlated with enactment of IPV reflect IPV perpetration or sexual entitlement in terms of content.

The role of social norms regarding violence against women and the culture of complicitness and acceptance of male perpetration of violence and harassment has sprung to the fore of national discussions. Men who perceive a higher peer or community prevalence of IPV perpetration or acceptance of this behavior may be more likely to perceive permission–or even experience greater pressure—to perpetrate violence themselves [17]. Unfortunately, we identified no measures of community norms and few measures of peer norms regarding IPV that met our inclusion criteria (N = 3 measures, from one high quality and one moderate quality study). While the three measures regarding peer norms around violence were less salient in predicting male perpetration than individual-level endorsement of norms [32,37], the studies were too few in number to draw definitive conclusions. This reflects a substantial research gap: how to best measure social norms around violence, or how to consider it in analyses or pathways of influence are outstanding questions.

Important to note are several methodological limitations to this review. First, it is possible that relevant studies were missed by our review. Reviewed data were most often observational in nature and extracted associations were cross-sectional, which precludes the ability to establish temporality. Studies with inappropriate or inadequate adjustment for confounders could result in a spurious finding, rather than differences attributable to specific scale types, content or construction. While we accounted for statistical control for covariates in our quality assessment, there is the potential for uncontrolled confounding to remain in reviewed studies. Additionally, while we assessed scale performance by considering associations established a priori by individual study authors, reporting deficiencies within articles may have masked non-significant results or other study limitations. Further, not all studies disaggregated perpetration by type of violence. There was insufficient sample size to stratify results by both the type of scale and the form of violence perpetrated. Therefore, this review assumes that perpetration of different forms of violence are interrelated and findings are synthesized across types of violence [48]. Future research to expand the evidence base on the role of masculinity in the perpetration of IPV could allow this assumption to be further explored. All data were self-reported and therefore vulnerable to recall and social desirability bias. As most of the studies were conducted among males, underreporting of IPV perpetration is a very real possibility, though whether a male who does not disclose perpetration would also bias his responses on a gender attitude scale to appear more equitable/progressive is an open question. Confirming male self-reports of violence perpetration with female reports of IPV experience should be further explored. Finally, no identified studies employed analytic techniques such as structural equation modeling, which may be better suited to measuring variables which may be co-determined or which can examine pathways of influence. Such techniques should be explored in future studies.

Our review also identified salient research gaps. Many identified scales were not validated or were excluded from this review because it was unclear what the scale measured (no scale items were reported). We also identified wide variation in how scales were labeled, defined and implemented. These measurement and reporting challenges make it difficult to ascertain patterns in the association between gender inequity measures and IPV perpetration. Despite these limitations, this review suggests that multi-item scales that are explicit in nature and reflect endorsement of violence against women or male sexual entitlement are more consistently associated, and therefore may be more salient to male perpetration of IPV. In contrast, single-item indicators, scales more general in nature, those which do not reference violence, tended to be less consistently associated with violence perpetration. Results from this study also suggest that validated scales and those which reflect acceptance of violence against women or male sexual entitlement tended to be more robust across settings and sample populations, and may be of practical utility in monitoring progress in preventing the perpetration of IPV. Further research is needed to understand how normative changes at the peer and community levels contribute to or sustain individual-level behavior change [4].

## Conclusion

This systematic review identified three major content areas of gender inequity measures commonly implemented in IPV research: views on gender roles/norms, endorsement of violence against women, and relationship power and control. We find that most measures reflected individual-level views or behaviors while few reflected normative influences operating at the peer level and no identified studies measured norms at the community or other level. Overall, we found that gender inequity measures were inconsistently associated with male perpetration of IPV. However, the relationship was sensitive to how such constructs were measured. Our findings suggest the importance of validated scales which include views on the acceptance of violence against women and male sexual entitlement in measuring determinants of male IPV perpetration. To move the field forward, we also argue for greater standardization of scale terminology in the field and further innovation and validation of scales that aim to capture gender inequitable norms, views, practices and relations. Longitudinal data which model an explicit and multi-level theory of behavior change would be useful for intervention design to identify what drives and what sustains change in IPV perpetration.

## Supporting information

S1 Table Sample key term search string used in PubMed, EconLit, SocIndex, POPline, and Women’s Studies International. (DOCX)

S2 Table PRISMA checklist. (DOC)
