# Supplementary material for: Gender, power, and violence: A systematic review of measures and their association with male perpetration of IPV
Source: PLoS One. 2018 Nov 29;13(11):e0207091. doi: 10.1371/journal.pone.0207091 (PMC6264844; doi:10.1371/journal.pone.0207091)
Supplement: S1 Table — (DOCX) [file pone.0207091.s001.docx]

**Supplemental Table 1:** Sample key term search string used in PubMed, EconLit, SocIndex, POPline, and Women’s Studies International.

(((((((((((measurement[Title/Abstract]) OR measuring[Title/Abstract]) OR measure[Title/Abstract]) OR measures[Title/Abstract])) OR ((((scale[Title/Abstract]) NOT "scale-up"[Title/Abstract])) OR index[Title/Abstract])))) OR ((indicator[Title/Abstract]) OR indicators[Title/Abstract])))) AND ((("2000/01/01"[Date - Publication] : "2015/12/31"[Date - Publication])) AND ((((((((((("spousal abuse") AND ((((attitude or attitudes))) OR (norm or norms))))) OR ((("partner violence") AND ((((attitude or attitudes))) OR (norm or norms))))) OR ((("wife beating") AND ((((attitude or attitudes))) OR (norm or norms)))))) OR (((((((((("decision-making") AND power)) AND sexual))))) OR (((((("decision-making") AND power)) AND (((((((husband) OR wife)) AND (("decision-making") AND power))))))) OR (((((((((((((((((((((((((("gender inequity") OR "gender equity")) OR (("gender attitude") OR "gender attitudes")) OR "gender beliefs") OR (("gender inequitable") OR "gender equitable")) OR "gender relations") OR "women's empowerment") OR "household decision-making") OR "gender empowerment") OR "relationship power") OR "women's agency") OR ((masculinity) OR femininity)) OR "partner control") OR (("gender role") OR "sex role")) OR ((("sexual pressures") OR "sexual pressure"))) OR "sexual self-efficacy") OR "sexual agency") OR "sexual power") OR "sexual subjectivity") OR "silencing the self") OR (("women's power in sexual and reproductive health"))))))) OR (((((("sexual relationship") OR "sexual relationships")) AND power)))) OR (((((((("safer sex") AND "self-efficacy")))) OR (((("sexual communication") AND "self-efficacy")))) OR (((("sexual decision-making") AND "self-efficacy")))) OR "condom self-efficacy")))
